# Circ_0000396 suppresses the proliferation and inflammation of rheumatoid arthritis synovial fibroblasts by targeting miR-574-5p/RSPO1 axis

**DOI:** 10.1186/s13018-023-04117-5

**Published:** 2023-09-22

**Authors:** Hongchao Yu, Jin Yang, Kun Chen, Wulin Kang, Fengfeng Zhu

**Affiliations:** 1 Department of Bone Disease, The Affiliated Hospital of Shaanxi University of Chinese Medicine, Xianyang, China; 2 Department of Trauma, The Affiliated Hospital of Shaanxi University of Chinese Medicine, Deputy 2 Weiyang West Road, Xianyang City, 712000 Shaanxi Province China

**Keywords:** Rheumatoid arthritis, Synovial fibroblasts, Circ_0000396, miR-574-5p, RSPO1

## Abstract

**Background:**

Circular RNAs (circRNAs) are important regulators on the onset and progression of rheumatoid arthritis (RA). Our purpose is to explore the role and underpin mechanism of circ_0000396 in RA progression.

**Methods:**

RA patients (*n* = 39) and healthy volunteers (*n* = 33) were recruited from the Affiliated Hospital of Shaanxi University of Chinese Medicine for the present work. Circ_0000396, microRNA-574-5p (miR-574-5p) and R-spondin 1 (RSPO1) RNA levels were analyzed by reverse transcription-quantitative polymerase chain reaction. Cell proliferation was analyzed by 3-(4,5-dimethyl-thiazol-2-yl)-2,5-diphenyltetrazolium bromide (MTT) assay, colony formation assay, and 5-ethynyl-2′-deoxyuridine (EDU) assay. Cell apoptosis was assessed by flow cytometry. Protein expression levels of proliferating cell nuclear antigen (PCNA), Cyclin D1, Cyclin E1, BCL2-associated × protein (Bax), B-cell lymphoma-2 (Bcl2), interleukin-1β (IL-1β), tumor necrosis factor-α (TNF-α) and RSPO1 were detected by western blot assay. Enzyme-linked immunosorbent assay (ELISA) was conducted to analyze the secretion of pro-inflammatory cytokines including IL-1β and TNF-α. The interaction between miR-574-5p and circ_0000396 or RSPO1 was confirmed by dual-luciferase reporter assay and RNA-pull down assay.

**Results:**

Circ_0000396 expression was notably down-regulated in RA patients compared with healthy controls. Circ_0000396 overexpression suppressed the proliferation and inflammatory response and triggered the apoptosis of RA synovial fibroblasts (RASFs), accompanied by decreases in PCNA, Cyclin D1, Cyclin E1, Bcl2, IL-1β and TNF-α protein expression and an increase in Bax protein expression. Circ_0000396 acted as a molecular sponge for miR-574-5p, and circ_0000396 overexpression-mediated protective effects on RASFs dysfunction were largely reversed by the introduction of miR-574-5p mimics. miR-574-5p interacted with the 3’ untranslated region (3’UTR) of RSPO1, and miR-574-5p negatively regulated RSPO1 expression in RASFs. Circ_0000396 could up-regulate the expression of RSPO1 by sponging miR-574-5p in RASFs. RSPO1 interference largely overturned circ_0000396 overexpression-mediated effects in RASFs.

**Conclusion:**

Circ_0000396 restrained the proliferation and inflammation and induced the apoptosis of RASFs by mediating miR-574-5p/RSPO1 axis, which provided novel potential targets for RA treatment.

**Supplementary Information:**

The online version contains supplementary material available at 10.1186/s13018-023-04117-5.

## Introduction

Rheumatoid arthritis (RA) is a systemic autoimmune disease that causes irreversible joint damage [1]. Accumulating articles demonstrated that RA synovial fibroblasts (RASFs) exert vital roles in RA pathology [2, 3]. RASFs are found to be similar to tumor cells in multiple biological phenotypes, including excessive proliferation, metastasis, and apoptosis resistance [4, 5]. Moreover, pro-inflammatory cytokines such as interleukin-1β (IL-1β) and tumor necrosis factor-α (TNF-α) activate immune response to contribute to RA progression [6]. Therefore, understanding the molecular mechanism of RASFs dysfunction is important for RA intervention and therapy.

It is reported that circular RNAs (circRNAs) are implicated in the pathological mechanism of multiple diseases, including cancers, cardiovascular diseases, and autoimmune diseases [7–9]. Accumulating evidence have demonstrated that circRNAs play vital regulatory roles in RA pathology. For instance, circ_ 0004712 is reported to facilitate the proliferation, migration and inflammatory response of RA fibroblast-like synoviocytes in RA [10]. Luo et al. demonstrated that circ_0002715 and circ_0035197 in peripheral blood are novel non-invasive bio-markers for RA patients [11]. Circ_0000396 is a newly discovered circRNA. A previous study reported that circ_0000396 is down-regulated in RA, and it restrains cell proliferation and inflammation in RASFs by targeting microRNA-203 (miR-203)/HMG-box transcription factor 1 (HBP1) axis [12]. In this study, the underpin mechanism of circ_0000396 in RA progression was further explored.

Short strand non-coding RNAs including microRNAs (miRNAs) and small interfering RNAs are related to the pathogenesis of tendon injuries and osteoarthritis [13–16]. In particular, miRNAs are another category of non-coding RNAs and are important regulators in RA pathology [17]. For example, miR-21 is reported to suppress the progression of RA in rats by regulating Wnt signaling [18]. It is well established that circRNAs can regulate gene expression by acting as miRNA sponges [19, 20]. miR-574-5p was predicted as a target of circ_0000396 through searching Circinteractome database. The functional association of circ_0000396 and miR-574-5p in RA pathology was investigated.

miRNAs can induce the degradation of target mRNAs by base-pairing with their 3’UTR [21]. In this study, targetscan database predicted the potential binding sequence of miR-574-5p on R-spondin 1 (RSPO1). RSPO1 expression is reported to be down-regulated in RA patients [22]. Here, we explored the functional correlation of miR-574-5p and RSPO1 in RA pathology.

We first analyzed circ_0000396 expression profile in RA. The biological role of circ_0000396 and its working mechanism were then explored by functional experiments.

## Materials and methods

### Clinical samples

RA patients (*n* = 39) and healthy volunteers (*n* = 33) were recruited from The Affiliated Hospital of Shaanxi University of Chinese Medicine for the expression detection of key molecules with the authorization by the Ethical Committee of The Affiliated Hospital of Shaanxi University of Chinese Medicine. 5 mL peripheral blood was collected from each participant undergoing routine examination and centrifuged to obtain serums at 2500 g for 10 min. Serum was stored at -80 °C until use. All the participants had signed the written informed consent. The diagnosis of RA was performed according to the 2010 European League against Rheumatism (EULAR) classification criteria/American College of Rheumatology (ACR) [23]. The inclusion criteria of RA patients: (1) meeting the above diagnostic criteria; (2) no history of malignancies and other serious diseases; (3) no treatment received within 3 months before admission. The exclusion criteria of RA patients: (1) patients have other rheumatic diseases; (2) patients with autoimmune diseases, tumors, or mental disorders; (3) patients with coronary heart disease, diabetes or other system diseases; (4) patients without complete medical records. Demographic characteristics of the study subjects are shown in Additional file 1: Table S1.

### Cell line

Human RASFs were isolated from synovial tissues that were collected from RA patients during total joint replacement surgery as previously reported [24]. RASFs at passage 3–8 were used for all the experiments. RASFs were maintained in Dulbecco’s modified Eagle’s medium (DMEM) (Invitrogen, Carlsbad, CA, USA) supplemented with 10% fetal bovine serum (FBS) (Gibco, Carlsbad, CA, USA).

### RNase R digestion

Total RNA samples containing circ_0000396 and its linear form solute carrier family 38 member 1 (SLC38A1) were treated with RNase R (100 μg/mL; Applied Biological Materials, Vancouver, Canada) for 20 min at 37 °C. Reverse transcription-quantitative polymerase chain reaction (RT-qPCR) was implemented to detect RNA levels.

### RT-qPCR

RASFs (5 × 10^6^) and serum samples were treated with TRIzol reagent (Thermo Fisher Scientific, Waltham, MA, USA) to isolate RNA. The first strand of complementary DNA (cDNA) for circRNA and mRNA was obtained using cDNA Reverse Transcription Kit (Thermo Fisher Scientific), and miRNA cDNA Synthesis Kit (Sangon Biotech, Shanghai, China) was utilized to synthesize cDNA of miRNA. qPCR reaction was proceeded with 2 × SYBR Green reagent (ABI, Carlsbad, CA, USA). The relative expression levels were normalized to glyceraldehyde 3-phosphate dehydrogenase (GAPDH) or small nuclear RNA U6 and evaluated by the 2^−ΔΔCt^ formula. All primers are displayed in Table 1.

**Table 1:** Primer sequences in RT-qPCR assay

Gene	Sequence (5′-3′)
circ_0000396	Forward: CATTATGGGCAGTGGGATTT Reverse: GTATGGCCTTCCAGGGTTTT
SLA38A1	Forward: GCTTTGGTTAAAGAGCGGGC Reverse: AGCTTGACACCCCTGTTAGC
miR-574-5p	Forward: TCGGCAGGTGAGTGTGTGTGTG Reverse: GCAGGGTCCGAGGTATTC
RSPO1	Forward: TGGCAAGGACTGGTGTTTGT Reverse: GTCACGTCAGCAGGGACTAC
GAPDH	Forward: TATGATGACATCAAGAAGGTGGT Reverse: TGTAGCCAAATTCGTTGTCATAC
U6	Forward: GCTTCGGCAGCACATATACTAAAAT Reverse: CGCTTCACGAATTTGCGTGTCAT

### Cell transfection

Circ_0000396 plasmid (circ_0000396), pLO-ciR vector (Vector), siRNA targeting RSPO1 (si-RSPO1), and si-NC were acquired from Genepharma (Shanghai, China), and mimics of miR-574-5p (miR-574-5p) and miR-NC were acquired from Ribobio (Guangzhou, China). Lipofectamine 3000 (Invitrogen) was utilized for cell transfection.

### 3-(4,5-dimethyl-thiazol-2-yl)-2,5-diphenyltetrazolium bromide (MTT) assay

RASFs were plated onto 96-well cell culture plates at 5000 cells/well, and were then reacted with 10 μL of MTT reagent (Beyotime, Shanghai, China) for 2–4 h the next day. A total of 100 μL of dimethyl sulfoxide (Sigma, St. Louis, MO, USA) was then added to each well to react with the insoluble crystals. The absorbance of each well at 570 nm was examined.

### Colony formation assay

Transfected RASFs were plated onto 12-well plates at 200 cells/well. Then, colonies were allowed to grow for 14 d, and the culture media was replenished every 4 d. The colonies were washed with phosphate-buffered saline (PBS), immobilized with 4% paraformaldehyde and then dyed with 0.5% crystal violet. The colony number of each well was counted.

### 5-ethynyl-2′-deoxyuridine (EDU) assay

Cell-Light™ EDU Apollo®567 Imaging Kit (Ribobio) was utilized to analyze cell proliferation ability. Transfected RASFs were incubated with 50 μM EDU reagent for 2 h. The nucleus of RASFs was marked with 2-(4-Amidinophenyl)-6-indolecarbamidine dihydrochloride (DAPI) (Sigma). Cell images were taken under a fluorescence microscope.

### Western blot assay

RASFs (8 × 10^5^) and serum samples were exposed to RIPA buffer to extract total proteins. Protein concentration was quantified as per the instruction of a bicinchoninic acid assay (BCA) protein assay kit (Pierce Biotechnology, Rockford, IL, USA). Afterward, the protein samples were loaded onto 10% separating gel and then shifted onto a polyvinylidene fluoride membrane (Millipore, Billerica, MA, USA). The membrane was incubated with the primary antibodies against proliferating cell nuclear antigen (PCNA) (ab29; 1:1000; Abcam, Cambridge, MA, USA), Cyclin D1 (ab16663; 1:200; Abcam), Cyclin E1 (ab33911; 1:1000; Abcam), BCL2-associated x protein (Bax) (ab32503; 1:5000; Abcam), B-cell lymphoma-2 (Bcl2) (ab32124; 1:1000; Abcam), IL-1β (ab216995; 1:1000; Abcam), TNF-α (ab183218; 1:1000; Abcam), RSPO1 (ab106556; 1:500; Abcam), and β-actin (ab8226, 1:1000; Abcam). Then, the membrane was incubated with HRP-conjugated secondary antibody (ab205718/ab205719; 1:5000; Abcam), and the protein bands were visualized by electrochemiluminescence. The intensities of protein bands were quantified using Image Lab analysis software (Bio-Rad, Hercules, CA, USA).

### Flow cytometry

Transfected RASFs were collected and dispersed in 500 μL of binding buffer. Then, Annexin V-fluorescein isothiocyanate (Annexin V-FITC) (Beyotime) and propidium iodide (PI) (Beyotime) were added to incubate with RASFs for 15 min in a dark room. The apoptosis rate was evaluated under a FC-500 flow cytometer (Beckman Coulter, Pasadena, CA, USA).

### Enzyme-linked immunosorbent assay (ELISA)

The secretion of IL-1β and TNF-α was analyzed by ELISA kit (Hengyuan, Shanghai, China). The absorbance at 450 nm was determined under a microplate reader (Thermo Fisher Scientific).

### Bioinformatics analysis

The interacted miRNAs of circ_0000396 and the mRNA targets of miR-574-5p were searched via Circinteractome (https://circinteractome.irp.nia.nih.gov/) and targetscan (http://www.targetscan.org/) databases, respectively.

### Dual-luciferase reporter assay

The wild-type (WT) or mutant (MUT) sequence of circ_0000396 or the 3’UTR of RSPO1 was sub-cloned to pmirGLO vector (Promega, Madison, WI, USA). The re-constructed plasmids were termed as circ_0000396-WT/MUT and RSPO1-3’UTR-WT/MUT. RASFs were transfected with 100 ng reporter and 50 nM small RNAs. The luciferase intensity was examined via a dual-luciferase assay system kit (Promega). Renilla luciferase activity was regarded as the control.

### RNA-pull down assay

The probe for miR-574-5p with biotin labeling was synthesized by GenePharma and was termed as Bio-miR-574-5p. Cell lysates were incubated with Bio-miR-574-5p or Bio-miR-NC and streptavidin agarose magnetic beads (Invitrogen). The enrichment of circ_0000396 was examined by RT-qPCR.

### Statistical analysis

All data were processed by GraphPad Prism 7 software and were expressed as the form of mean ± standard deviations (SD). The differences were analyzed by Student’s *t*-test or one-way analysis of variance (ANOVA) followed by Tukey’s test. *P* < 0.05 indicated a statistically significant difference.

## Results

### Circ_0000396 expression is down-regulated in RA patients

**Fig. 1:**
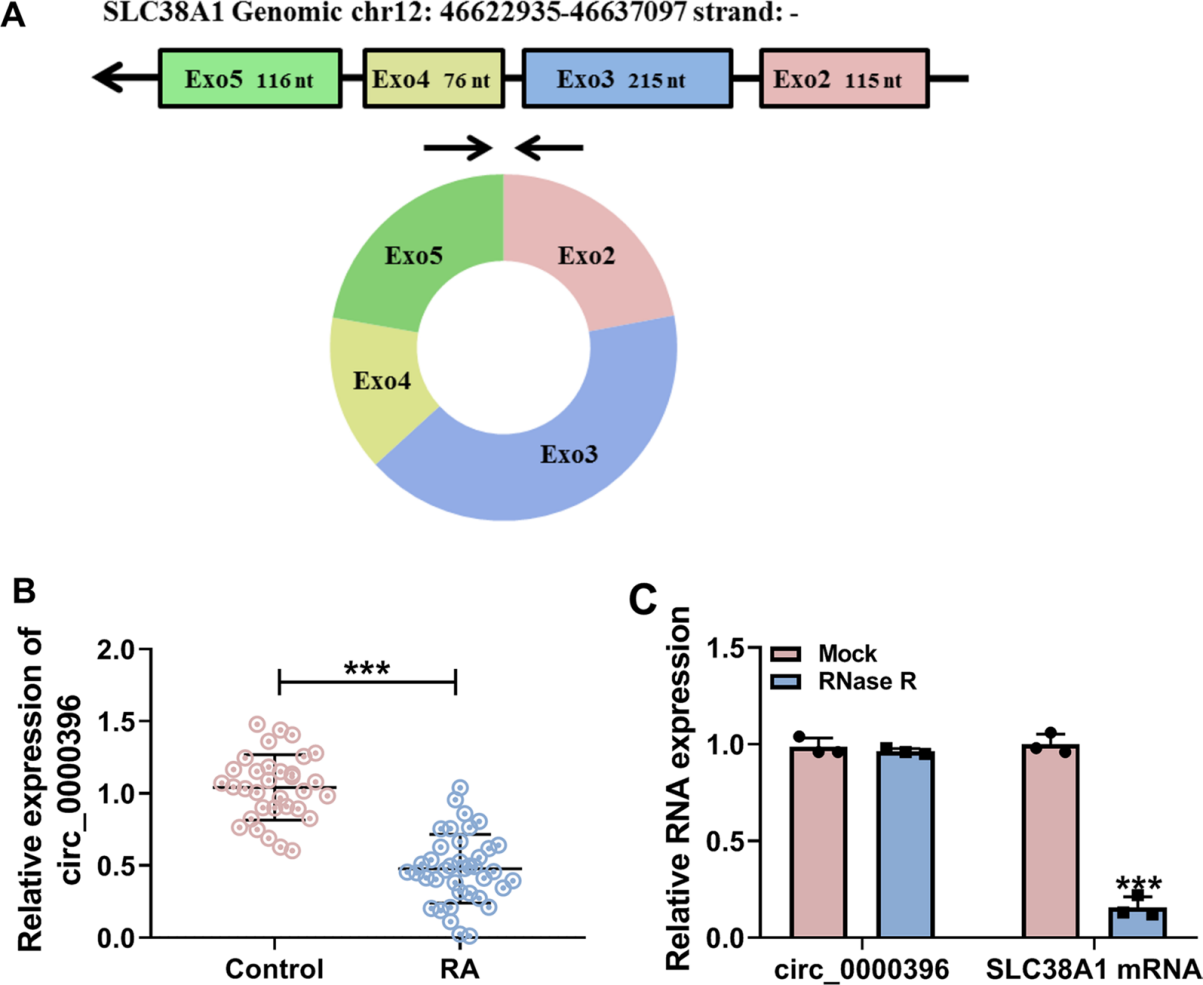
Circ_0000396 expression is down-regulated in RA patients. **A** Circ_0000396 is derived from exon 2–5 of the SLC38A1 gene. **B** RT-qPCR was conducted to determine the expression of circ_0000396 in the serum samples of RA patients (*n* = 39) and healthy volunteers (*n* = 33). **C** Exonuclease RNase R was used to analyze the stability of circ_0000396, and its linear form SLC38A1 mRNA acted as the control. ***P < 0.001

Circ_0000396 consists of exon 2–5 which derived from the host gene SLC38A1 (Fig. 1A). To explore the expression pattern of circ_0000396 in RA, RT-qPCR was implemented. Circ_0000396 expression was notably reduced in the serum samples of RA patients (*n* = 39) compared with non-RA controls (*n* = 33) (Fig. 1B). The stability of circ_0000396 was tested using exonuclease RNase R. Circ_0000396 was resistant to RNase R, while its linear form SLA38A1 was sensitive to RNase R (Fig. 1C), suggesting that circ_0000396 had potential as a bio-marker. These findings suggested that the level of circ_0000396 might be associated with RA progression.

### Circ_0000396 overexpression suppresses the proliferation and inflammation and induces the apoptosis of RASFs

**Fig. 2:**
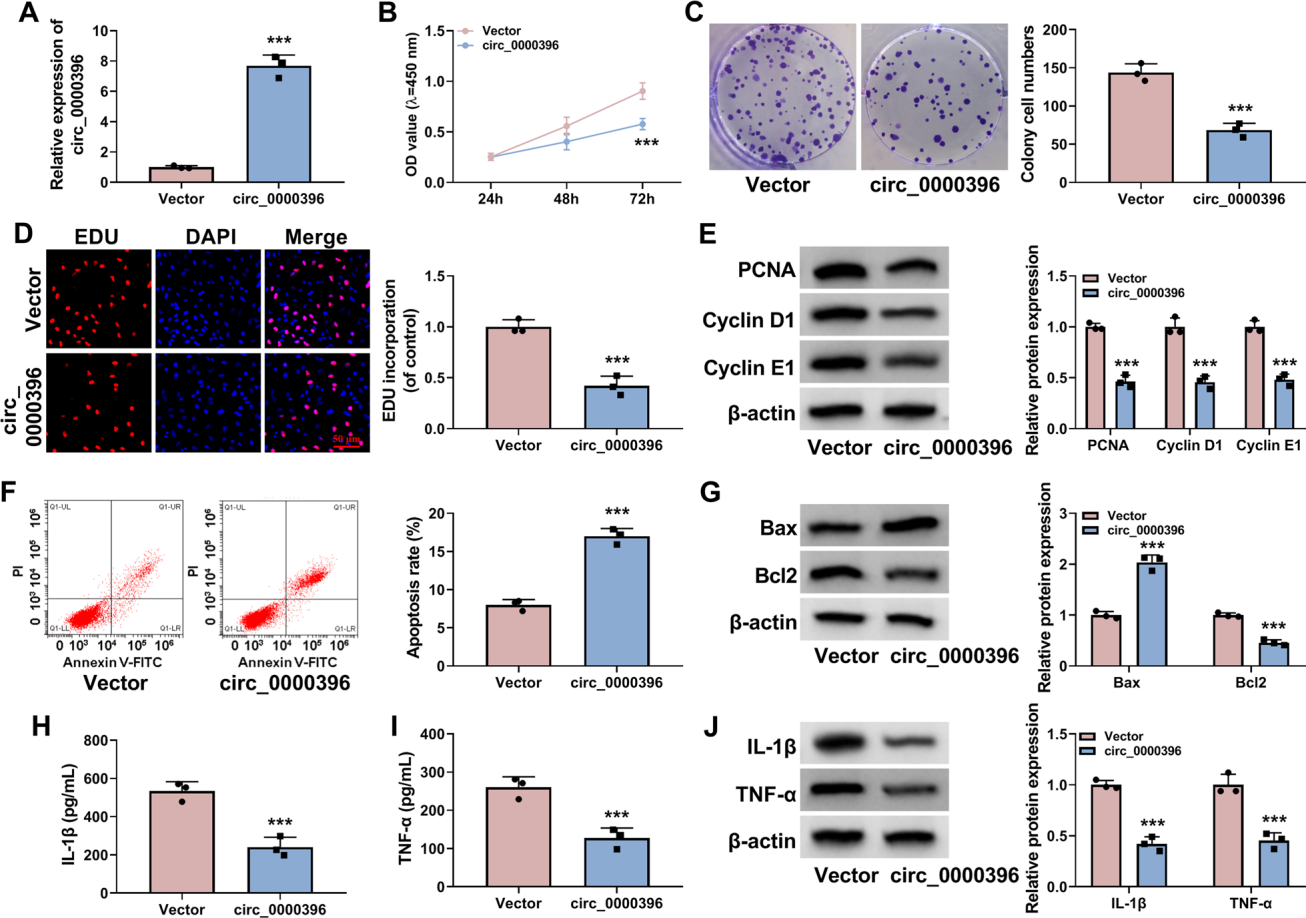
Circ_0000396 overexpression suppresses the proliferation and inflammation and induces the apoptosis of RASFs. **A**–**J** RASFs were transfected with Vector or circ_0000396 plasmid. **A** RT-qPCR was conducted to determine the expression of circ_0000396 in transfected RASFs. **B** MTT assay was performed to analyze the proliferation ability of transfected RASFs. **C** Colony formation assay was conducted to assess the proliferation capacity of transfected RASFs. **D** EDU assay was carried out to analyze the proliferation ability of transfected RASFs. **E** Western blot assay was implemented to examine the protein levels of PCNA, Cyclin D1, and Cyclin E1 in transfected RASFs. **F** The apoptosis rate of RASFs was analyzed by flow cytometry. **G** The protein levels of Bax and Bcl2 were determined by Western blot assay. **H** and **I** ELISA assay was conducted to analyze the release of pro-inflammatory cytokines (IL-1β and TNF-α). **J** The protein levels of IL-1β and TNF-α were examined by Western blot assay. ***P < 0.001

To analyze the biological role of circ_0000396 in RASFs, circ_0000396 was overexpressed in RASFs by transfecting with its ectopic expression plasmid. The overexpression efficiency of circ_0000396 plasmid was obvious in RASFs (Fig. 2A). The results of MTT assay, colony formation assay, and EDU assay together demonstrated that circ_0000396 overexpression restrained the proliferation of RASFs (Fig. 2B–D). Furthermore, Western blot assay showed that circ_0000396 overexpression reduced the protein levels of PCNA, Cyclin D1 and Cyclin E1 in RASFs (Fig. 2E), further suggesting that circ_0000396 overexpression inhibited cell proliferation in RASFs. Flow cytometry displayed that cell apoptosis rate was notably elevated by the overexpression of circ_0000396 (Fig. 2F). Moreover, Western blot assay revealed that circ_0000396 up-regulation elevated the level of pro-apoptotic protein Bax and reduced the level of anti-apoptotic protein Bcl2 (Fig. 2G), further indicating that circ_0000396 overexpression induced the apoptosis of RASFs. The release of pro-inflammatory cytokines (IL-1β and TNF-α) was suppressed by the overexpression of circ_0000396 compared with Vector group (Fig. 2H and I). By contrast with the Vector group, circ_0000396 overexpression reduced the protein levels of IL-1β and TNF-α in RASFs (Fig. 2J). Overall, circ_0000396 overexpression inhibited the proliferation and inflammation and induced the apoptosis of RASFs.

### miR-574-5p is a direct target of circ_0000396 in RASFs

**Fig. 3:**
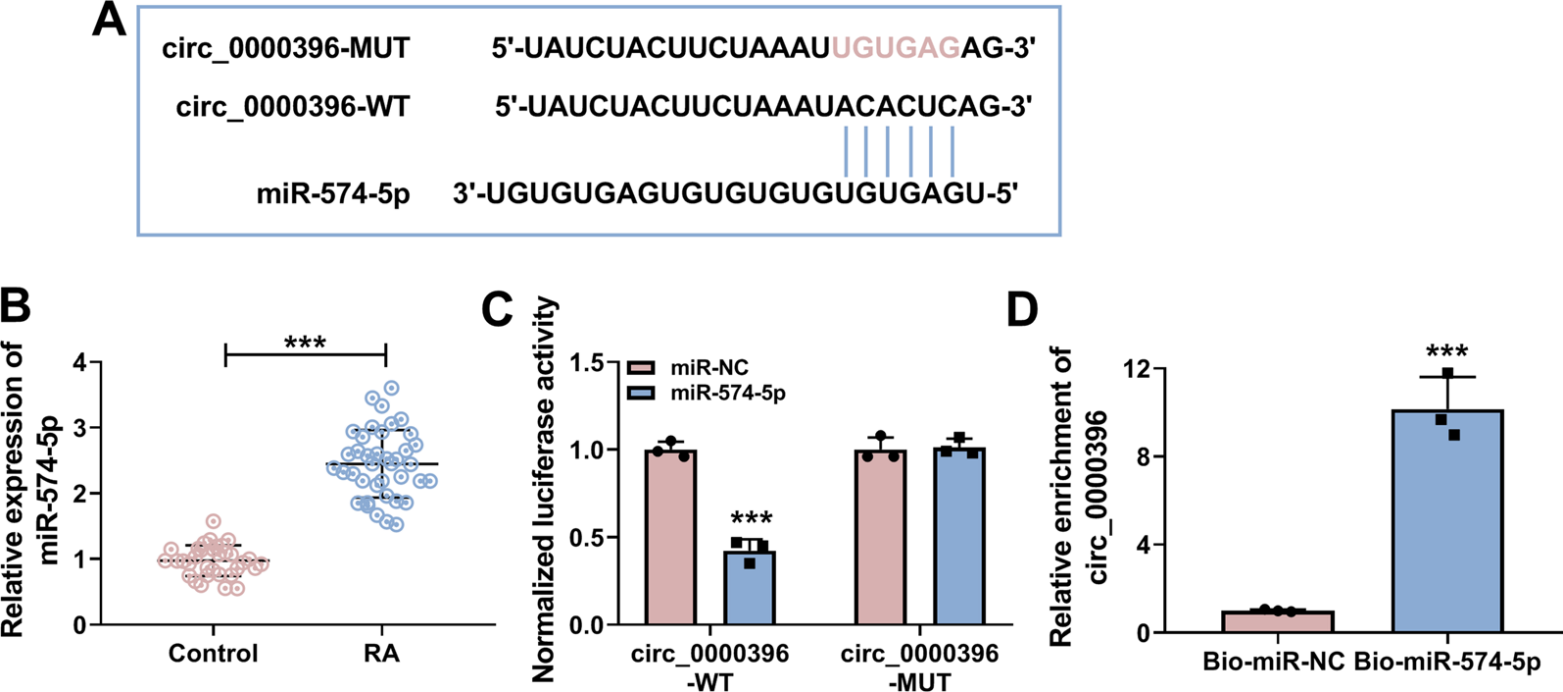
miR-574-5p is a direct target of circ_0000396 in RASFs. **A** The interacted miRNAs of circ_0000396 were predicted by bioinformatics software Circinteractome. miR-574-5p was predicted to be a possible target of circ_0000396, and their putative binding sites were shown. **B** RT-qPCR was conducted to determine the expression of miR-574-5p in the serum samples of RA patients (*n* = 39) and healthy volunteers (*n* = 33). **C** Dual-luciferase reporter assay was conducted to verify the interaction between circ_0000396 and miR-574-5p in RASFs. **D** RNA-pull down assay was implemented to confirm the target relation between circ_0000396 and miR-574-5p. ***P < 0.001

It has been reported that circRNAs can act as miRNA sponges to regulate gene expression and cellular biological phenotypes [25]. To investigate the underpin mechanism behind the role of circ_0000396 on RASFs dysfunction, the interacted miRNAs of circ_0000396 were predicted using bioinformatics software Circinteractome. The putative binding sites between circ_0000396 and miR-574-5p were shown in Fig. 3A. miR-574-5p expression was significantly up-regulated in RA patients relative to healthy controls (Fig. 3B). Afterward, dual-luciferase reporter assay and RNA-pull down assay were performed to verify the interaction between circ_0000396 and miR-574-5p. The luciferase activity of wild-type plasmid (circ_0000396-WT) was markedly reduced by the overexpression of miR-574-5p (Fig. 3C), suggesting the direct interaction between circ_0000396 and miR-574-5p in RASFs. The results of RNA-pull down assay showed that circ_0000396 was notably enriched in Bio-miR-574-5p group compared with Bio-miR-NC group (Fig. 3D), indicating that circ_0000396 bound to miR-574-5p in RASFs. These results demonstrated that miR-574-5p was a direct target of circ_0000396 in RASFs.

### Circ_0000396 regulates the biological behaviors of RASFs partly by sponging miR-574-5p

**Fig. 4:**
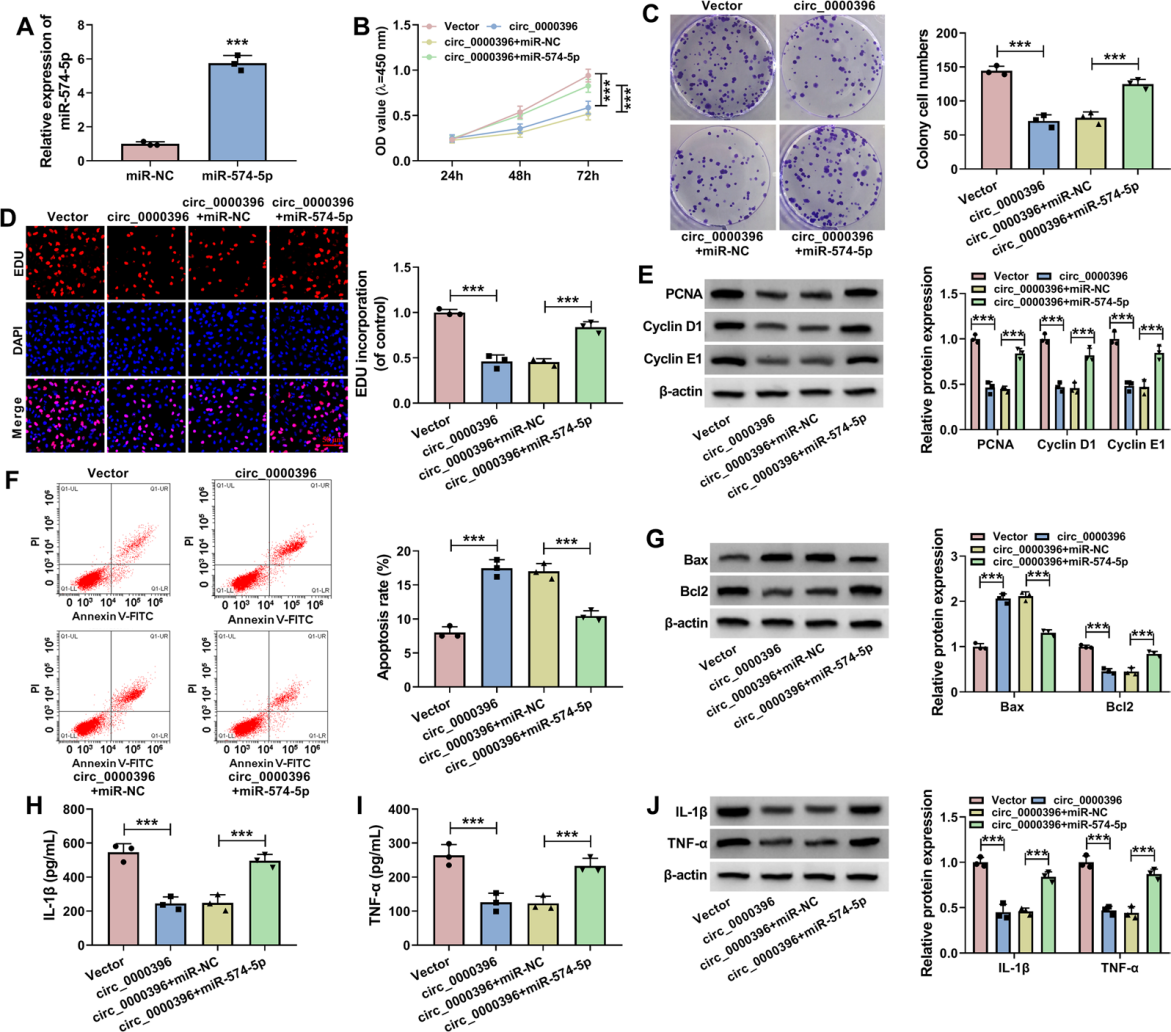
Circ_0000396 regulates the biological behaviors of RASFs partly by sponging miR-574-5p. **A** RT-qPCR was conducted to examine the level of miR-574-5p in RASFs transfected with miR-NC or miR-574-5p. **B**–**J** RASFs were transfected with circ_0000396 plasmid alone or together with miR-574-5p mimics. **B** MTT assay was conducted to analyze the proliferation ability of RASFs. **C** Colony formation assay was performed to assess the proliferation ability of RASFs. **D** EDU assay was implemented to analyze the proliferation ability of RASFs. **E** Western blot assay was conducted to measure the protein expression of PCNA, Cyclin D1, and Cyclin E1 in transfected RASFs. **F** Flow cytometry was conducted to analyze the apoptosis rate of transfected RASFs. **G** The protein levels of Bax and Bcl2 were detected by Western blot assay. **H** and **I** The concentrations of pro-inflammatory cytokines (IL-1β and TNF-α) in the culture supernatant of RASFs were analyzed by ELISA assay. **J** Western blot assay was carried out to measure the protein expression of IL-1β and TNF-α in transfected RASFs. **P < 0.01, ***P < 0.001

We explored whether miR-574-5p was implicated in the effects of circ_0000396 in RASFs, and compensation experiments were conducted. As expected, transfection with miR-574-5p mimics significantly up-regulated miR-574-5p level in RASFs (Fig. 4A). We found that circ_0000396 overexpression-induced suppressive effect on the proliferation of RASFs was largely overturned by miR-574-5p overexpression (Fig. 4B–E). Cell apoptosis induced by circ_0000396 overexpression was largely attenuated by the addition of miR-574-5p mimics (Fig. 4F and G). Circ_0000396 overexpression-induced inhibitory effect on the inflammation of RASFs was largely reversed by the introduction of miR-574-5p mimics (Fig. 4H–J). Taken together, circ_0000396 suppressed the proliferation and inflammatory response and induced the apoptosis of RASFs largely by interacting with miR-574-5p.

### RSPO1 is a direct target of miR-574-5p in RASFs

**Fig. 5:**
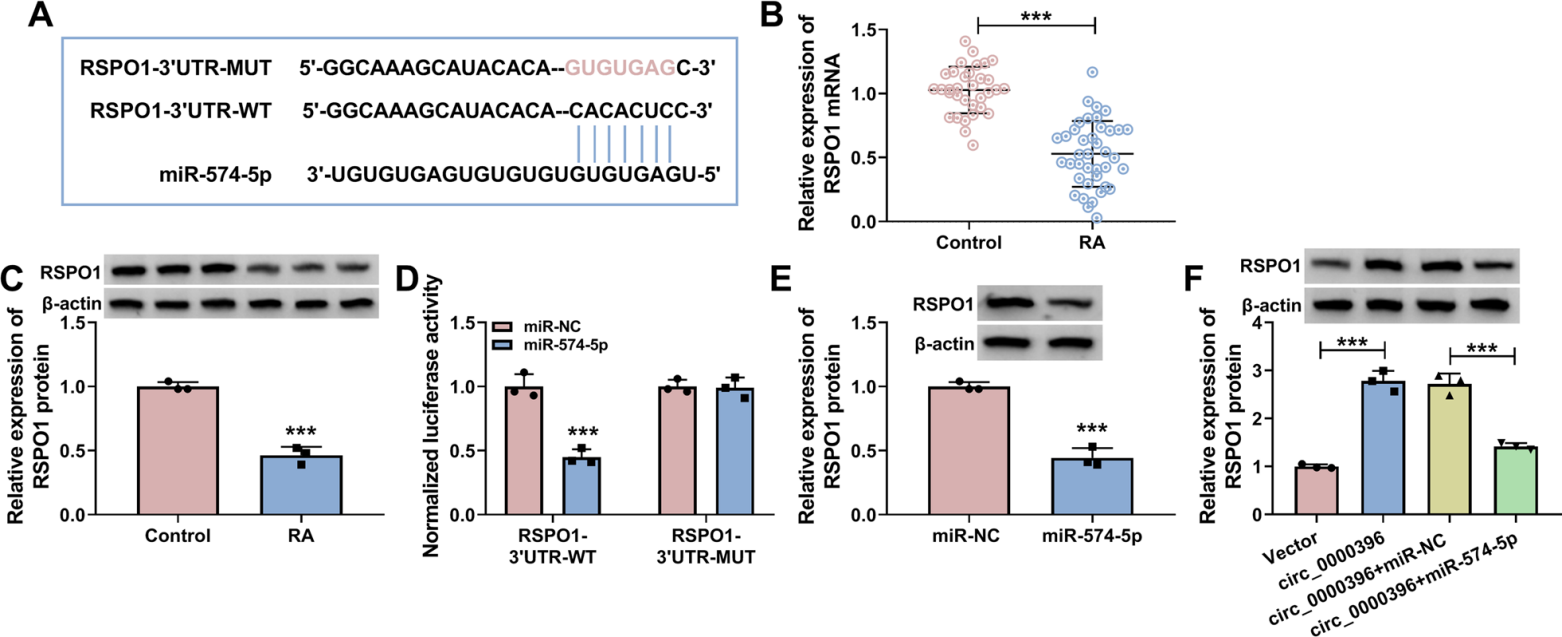
RSPO1 is a direct target of miR-574-5p in RASFs. **A** The putative binding sites between miR-574-5p and the 3’UTR of RSPO1 were predicted by bioinformatics software targetscan. **B** RT-qPCR was implemented to detect the mRNA level of RSPO1 in the serum samples of RA patients (*n* = 39) and healthy volunteers (*n* = 33). **C** Western blot assay was conducted to analyze the protein level of RSPO1 in the serum samples of RA patients and healthy volunteers. **D** The interaction between miR-574-5p and RSPO1 was tested by dual-luciferase reporter assay. **E** Western blot assay was conducted to measure the protein level of RSPO1 in RASFs transfected with miR-NC or miR-574-5p. **F** The protein expression of RSPO1 was determined in RASFs transfected with circ_0000396 alone or together with miR-574-5p by Western blot assay. ***P < 0.001

Through searching bioinformatics software targetscan, RSPO1 was predicted as a target of miR-574-5p (Fig. 5A). The expression pattern of RSPO1 in RA was explored. The mRNA and protein expression of RSPO1 was markedly down-regulated in RA patients relative to that in healthy controls (Fig. 5B and C). Dual-luciferase reporter assay uncovered that miR-574-5p overexpression significantly reduced the luciferase activity of wild-type plasmid (RSPO1-3’UTR-WT) rather than mutant plasmid (RSPO1-3’UTR-MUT) (Fig. 5D), indicating the interaction between miR-574-5p and RSPO1 in RASFs. miR-574-5p overexpression notably reduced the protein expression of RSPO1 in RASFs (Fig. 5E), suggesting the negative relation between miR-574-5p and RSPO1 in RASFs. Circ_0000396 overexpression up-regulated the protein level of RSPO1, and the addition of miR-574-5p reduced its protein level (Fig. 5F), suggesting that circ_0000396 positively regulated RSPO1 expression by sponging miR-574-5p in RASFs. Overall, RSPO1 was a target of miR-574-5p, and it was regulated by circ_0000396/miR-574-5p axis in RASFs.

### RSPO1 interference partly reverses circ_0000396 overexpression-induced effects in RASFs

**Fig. 6:**
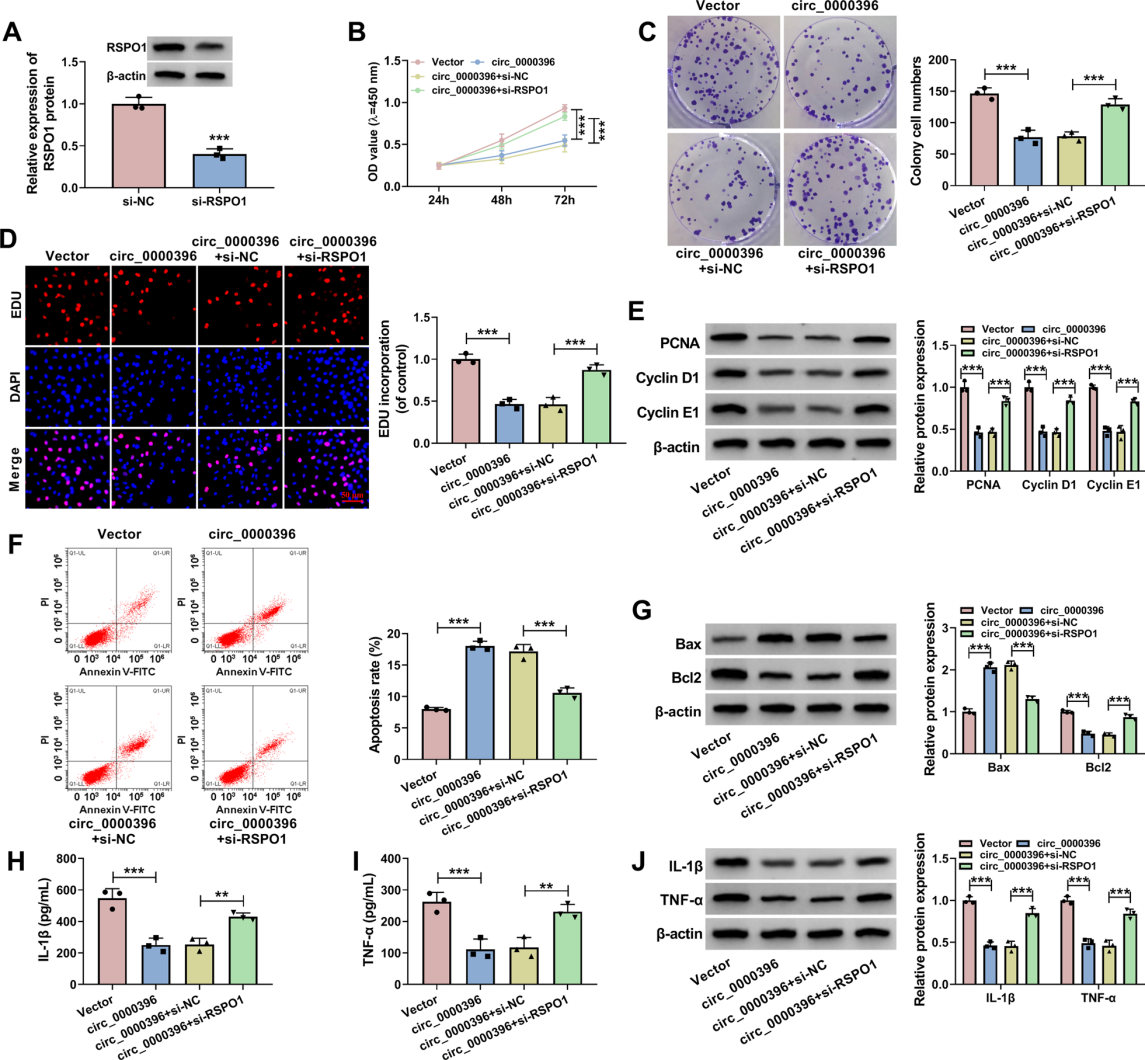
RSPO1 interference partly reverses circ_0000396 overexpression-induced effects in RASFs. **A** Western blot assay was conducted to determine the protein expression of RSPO1 in RASFs transfected with si-NC or si-RSPO1. (B-J) RASFs were transfected with circ_0000396 plasmid alone or together with si-RSPO1. **B** Cell proliferation was analyzed by MTT assay. **C** Cell proliferation was analyzed by colony formation assay. **D** EDU assay was conducted to assess the proliferation ability of RASFs. **E** The protein levels of PCNA, Cyclin D1 and Cyclin E1 were determined by Western blot assay. **F** The apoptosis rate of RASFs was assessed by flow cytometry. **G** The protein levels of Bax and Bcl2 were examined by Western blot assay. **H** and **I** Cell inflammation was analyzed by ELISA assay. **J** The protein levels of IL-1β and TNF-α were examined by Western blot assay. **P < 0.01, ***P < 0.001

To explore whether circ_0000396 functioned in RASFs by regulating RSPO1, we conducted compensation experiments by dividing RASFs into the following four groups: Vector, circ_0000396, circ_0000396 + si-NC, and circ_0000396 + si-RSPO1. Transfection with si-RSPO1 markedly reduced the protein level of RSPO1 in RASFs (Fig. 6A), suggesting the successful transfection. Circ_0000396-mediated proliferation arrest, apoptosis induction, and inflammation suppression in RASFs were all largely reversed by the introduction of si-RSPO1 (Fig. 6B–J). Taken together, circ_0000396 played a role in RASFs partly by up-regulating RSPO1.

## Discussion

Accumulating articles have demonstrated that RA, an autoimmune disease with joint synovial inflammatory response as the pathological characteristic, is closely related to the aberrant hyperplasia of RASFs [3, 26]. CircRNAs are reported to be pivotal regulators in multiple human diseases [27, 28]. Moreover, increasing studies reported that circRNAs are implicated in the initiation and development of several autoimmune diseases, containing RA [29–31]. We observed that circ_0000396 was notably down-regulated in RA patients compared with healthy volunteers, which was in agreement with previous studies [12, 32]. The excessive proliferation and insufficient apoptosis of RASFs may result in bone destruction [33]. Gain-of-function experiments displayed that circ_0000396 accumulation hampered cell proliferation and induced cell apoptosis in RASFs. Pro-inflammatory cytokines such as IL-1β and TNF-α can stimulate cell proliferation of RASFs to produce IL-6, chemotactic factor, matrix metalloproteins (MMPs), and rostaglandin, eventually resulting in cartilage injury and the initiation of RA [34]. We found that the secretion of inflammation-associated cytokines was notably suppressed by the overexpression of circ_0000396, suggesting that circ_0000396 overexpression blocked cell inflammatory response in RASFs. These results together demonstrated that circ_0000396 suppressed RA development through suppressing the proliferation and inflammation and inducing the apoptosis of RASFs.

It is extensively established that circRNAs can serve as miRNA sponges to modulate gene expression and cellular biological behaviors [19]. miRNAs exert multifunctional roles in the initiation and progression of RA. For instance, miR-124a is reported to suppress the proliferation and invasion abilities of RASFs to hamper RA progression [35]. miR-613 is reported to block cell growth and invasion abilities and trigger cell apoptosis in RASFs by reducing the expression of dickkopf-related protein 1 (DKK1) [36]. We verified miR-574-5p as a novel miRNA target of circ_0000396. miR-574-5p is reported to induce the differentiation of osteoclasts by targeting toll-like receptor (TLR) 7/8 in RA [37]. However, its biological role in RASFs remains to be clarified. We observed that miR-574-5p abundance was elevated in RA patients relative to healthy volunteers. Circ_0000396 overexpression-induced protective effects on RASFs dysfunction were partly offset by miR-574-5p mimics, proving that circ_0000396 overexpression hampered RA development partly by reducing miR-574-5p abundance.

Generally, miRNAs can regulate multiple cellular phenotypes by base-pairing with 3’UTR of mRNAs to induce their degradation or translational repression [38]. Previous articles have identified many mRNA targets of miR-574-5p, including at-rich interaction domain 3A (ARID3A) [39], forkhead box N3 (FOXN3) [40], and caspase activation and recruitment domain 3 (CARD3) [41]. We confirmed RSPO1 as a novel target of miR-574-5p. RSPO1 is reported to directly regulate osteoblast differentiation and maturation [42]. Furthermore, a previous study demonstrated that RSPO1 played a joint protective role against inflammatory arthritis in TNF-α transgenic mouse model [43]. RSPO1 expression is reported to be down-regulated in RA patients [22]. Consistent with former study [22], we found that RSPO1 level was decreased in the serum of RA patients. Moreover, we observed that RSPO1 was inversely modulated by miR-574-5p in RASFs. RSPO1 was regulated by circ_0000396/miR-574-5p in RASFs. Circ_0000396 overexpression-mediated protective effects on RASFs dysfunction were largely reversed by the silence of RSPO1, indicating that circ_0000396 exerted a protective role in RASFs partly by up-regulating RSPO1.

**Fig. 7:**
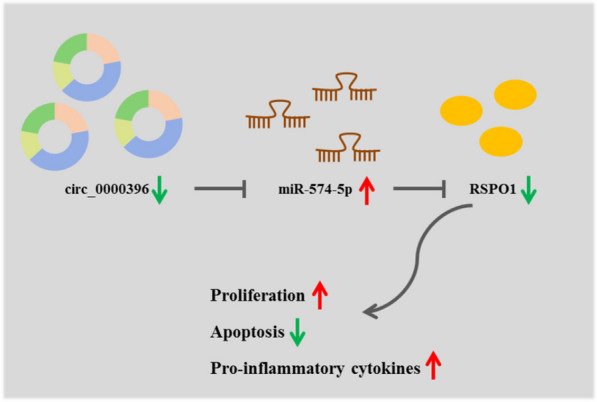
Schematic model showing the role of circ_0000396 in regulating the proliferation, apoptosis, and inflammation of RASFs

Taken together, our findings demonstrated that circ_0000396 blocked the proliferation and inflammation and facilitated the apoptosis of RASFs by targeting miR-574-5p/RSPO1 axis (Fig. 7). However, there were several limitations of our study that should be noted. Only one cell line was used in this study. Furthermore, animal model needs to be used to verify the effects of circ_0000396 in vivo for clinical application.

### Supplementary Information

**Additional file 1.** **Table S1**: Baseline features and clinical parameters of the study subjects.

## Data Availability

Data sharing not applicable to this article as no datasets were generated or analyzed during the current study.
