# Supplementary material for: Circ_0000396 suppresses the proliferation and inflammation of rheumatoid arthritis synovial fibroblasts by targeting miR-574-5p/RSPO1 axis
Source: J Orthop Surg Res. 2023 Sep 22;18:718. doi: 10.1186/s13018-023-04117-5 (PMC10514958; doi:10.1186/s13018-023-04117-5)
Supplement: Supplementary file 1 — Additional file 1. Table S1: Baseline features and clinical parameters of the study subjects. [file 13018_2023_4117_MOESM1_ESM.docx]

| Characteristics | Control | RA |
| --- | --- | --- |
| Number | 33 | 39 |
| Gender (male/female) | 10/23 | 9/30 |
| Age (years) | 57.34±8.46 | 59.41±8.04 |
| BMI (kg/m^2^) | 25.8±3.8 | 26.2±3.6 |
| RF (IU/mL) | 186.45±165.42 | <9.5 |
| CRP (mg/L) | 13.56±11.48 | 0.98±0.35 |
| ESR (mm/h) | 42.51±22.34 | 8.63±2.67 |
| Anti-CCP-Ab (RU/mL) | 485.26±275.44 | 16.74±1.82 |
| DAS28 | - | 3.56±0.72 |

**Table S1 Baseline features and clinical parameters of the study subjects**

RA, Rheumatoid arthritis; BMI: body mass index; RF, rheumatoid factor; CRP, C-reactive protein; ESR, erythrocyte sedimentation rate; anti-CCP-Ab, anti-cyclic citrullinated peptide antibody; DAS28: disease activity score (Disease activity scores below 3.2 indicate low disease activity, while 3.2–5.1 and above 5.1 indicate moderate and high disease activity, separately).
